# Echiniscidae from the Sierra Nevada de Santa Marta, Colombia, new records and a new species of *Bryodelphax* Thulin, 1928 (Tardigrada)

**DOI:** 10.3897/zookeys.703.12537

**Published:** 2017-09-27

**Authors:** Oscar Lisi, Anisbeth Daza, Rosana Londoño, Sigmer Quiroga

**Affiliations:** 1 Grupo de Investigación MIKU, Facultad de Ciencias Básicas, Universidad del Magdalena, Carrera 32 No 22-08, Santa Marta D.T.C.H., Colombia; 2 Programa de Biología, Facultad de Ciencias Básicas, Universidad del Magdalena, Carrera 32 No 22-08, Santa Marta D.T.C.H., Colombia; 3 Dipartimento di Scienze Biologiche, Geologiche e Ambientali, Sezione di Biologia Animale “Marcello La Greca”, Università di Catania, Via Androne 81, 95124 Catania, Italy

**Keywords:** Biodiversity, *Bryodelphax kristenseni* sp. n., *Echiniscus*, Heterotardigrada, water bear

## Abstract

Three species of *Echiniscus* are recorded for the first time from Colombia: *Echiniscus dariae*, *Echiniscus kofordi*, and *Echiniscus perarmatus*. In addition, the description of the new species *Bryodelphax kristenseni* **sp. n.**, is mainly based on the presence of ten paired plus two unpaired granularly sculptured ventral plates, the dorsal plate ornamentation with superficial irregular pores, no spine on the anterior legs, and the hind legs without papillae or dentate collar.

## Introduction

To date, only ten species of Echiniscidae Thulin, 1928, have been reported from Colombia: *Echiniscus bigranulatus* Richters, 1908a, *Echiniscus blumi* Richters, 1903 *sensu lato*, *Echiniscus madonnae* Michalczyk & Kaczmarek, 2006, *Echiniscus quadrispinosus* Richters, 1902 *sensu lato*, *Echiniscus spiniger* Richters, 1904, *Echiniscus testudo* (Doyère, 1840), *Echiniscus virginicus* Riggin, 1962, *Echiniscus wendti* Richters, 1903, *Pseudechiniscus novaezeelandiae* (Richters, 1908b) *sensu lato*, and *Pseudechiniscus suillus* (Ehrenberg, 1853) (Meyer 2013, Lisi et al. 2014, Kaczmarek et al. 2015). In the present study, material deposited in the “Centro de Colecciones Biológicas de la Universidad del Magdalena” collected between 2011 and 2012 from different localities in the Sierra Nevada de Santa Marta (Colombia) was examined. In this material new records of *Echiniscus* C.A.S. Schultze, 1840, and a new species of *Bryodelphax* Thulin, 1928, were found which are described in this paper.

## Materials and methods

This survey was based on tardigrade specimens deposited in the Centro de Colecciones Biológicas de la Universidad del Magdalena under the catalogue acronym CBUMAG:TAR. The material was collected between 2011 and 2012, from different localities (San Lorenzo, Bella Vista, and Medium basin of Garupal River) in the Sierra Nevada de Santa Marta, Colombia, from 543 and 2,200 m a.s.l. All specimens were preserved on slides in Hoyer’s medium.

Tardigrades were examined using a Phase Contrast Microscope (PCM) Zeiss Axiolab A1 with an adapted digital camera Zeiss AxioCam ERc 5s used for the photographic records, and a Differential Interference Contrast Microscope (DIC) Zeiss Axio Scope A1. The measurements were acquired with the software Zeiss AxioVision SE64. The *sc* ratio is the ratio of the length of a given structure to the length of the scapular plate (Fontoura and Morais 2011). The configuration of ventral plates is indicated in accordance with Kaczmarek et al. (2012). Identification was based on morphological characters, using Ramazzotti and Maucci (1983) for species described before 1983, and literature containing the original descriptions of several species: Murray (1907), Schuster and Grigarick (1966), Pilato (1974), Pilato and Lisi (2003), Kaczmarek and Michalczyk (2004), Kaczmarek and Michalczyk (2010), Kristensen et al. (2010), Pilato et al. (2010). We also compared our material with the holotypes of *Echiniscus walteri* Pilato & Lisi, 2003 and *E. kofordi* Schuster & Grigarick, 1966.

For evaluations at genus level regarding *Bryodelphax*, the following material was examined from the Pilato and Binda collection: *Bryodelphax brevidentatus* Kaczmarek, Michalczyk & Degma, 2005 (paratype, slide No. 5386), *Bryodelphax meronensis* Pilato, Lisi & Binda, 2010 (holotype, slide No. 5350 and a paratype, slide No. 5347), *Bryodelphax parvulus* Thulin, 1928 (from Israel, slide No. 5348; from northern Italy, slide Nos. 1288, 1290–91; from Morocco, slide No. 1280; from central Sicily, slide No. 1880, and from Ustica island, about 60 km north of Sicily, slide No. 1296), *Bryodelphax mateusi* (Fontoura, 1982) (holotype, slide No. 5062).

## Results

### Class: Heterotardigrada Marcus, 1927

#### Order: Echiniscoidea Richters, 1926

##### Family: Echiniscidae Thulin, 1928

###### Genus: *Echiniscus* C.A.S. Schultze, 1840

####### Echiniscus dariae

Taxon classificationAnimalia EchiniscoideaEchiniscidae

Kaczmarek & Michalczyk, 2010

######## Material examined.

21 specimens, CBUMAG:TAR:00068 (1 specimen), 00085 (11 specimens), 00098 (2 specimens), 00099 (2 specimens), 00101 (1 specimen), 00102 (2 specimens), 00103 (2 specimens). Microhabitat: mixture of a moss from the family Meesiaceae and lichens of the genera *Hypotrachyna*, *Usnea*, *Parmotrema*, *Parmelinopsis*, growing on tree trunks. Localities: San Lorenzo, Sierra Nevada de Santa Marta, 11°06'20.0"N, 74°03'54.4"W, 1930 m a.s.l, and Bella Vista Sierra Nevada de Santa Marta, 11°05'47.8"N, 74°05'04.4"W, 2200 m a.s.l.

######## Remarks.

The morphological features of the specimens correspond with the description of *E. dariae* (Kaczmarek & Michalczyk, 2010), a species that has only been reported for the Neotropical region; with the type locality of Costa Rica, and Peru (Kaczmarek and Michalczyk 2010, Kaczmarek et al. 2014).

This is the first record of this species for Colombia.

####### Echiniscus kofordi

Taxon classificationAnimalia EchiniscoideaEchiniscidae

Schuster & Grigarick, 1966

######## Material examined.

11 specimens, CBUMAG:TAR:00143 (5 specimens), 00144 (6 specimens). Microhabitat: lichen from the genus *Parmotrema* growing on a tree trunk. Locality: Medium basin of Garupal River, Sierra Nevada de Santa Marta, 10°13'48.4"N, 073°48'01.5"W, 543 m a.s.l.

######## Remarks.

These specimens were compared with the holotypes of *E. walteri* Pilato & Lisi, 2003 and *E. kofordi* Schuster & Grigarick, 1966 deposited in the Binda and Pilato collection (Catania, Italy), concluding that the specimens corresponded perfectly with *E. kofordi*. This species has a wide distribution; with the type locality Santa Cruz Island (Galápagos Islands, Ecuador), it has been recorded from India (Andaman Islands), United States, Mexico, Costa Rica, and Venezuela (Grigarick et al. 1983, Meyer 2013). Due to the disjunct geographical distribution reported for this species, it is suspected that the Indian record might refer to another similar species.

This identification provides the first record for Colombia.

####### Echiniscus perarmatus

Taxon classificationAnimalia EchiniscoideaEchiniscidae

Murray, 1907

######## Material examined.

21 specimens, CBUMAG:TAR:00098 (7 specimens), 00099 (10 specimens), 00101 (4 specimens). Microhabitat: lichen from the genus *Parmotrema*, growing on a tree trunk. Localities: Bella Vista, Sierra Nevada de Santa Marta, 11°05'47.8"N, 74°05'04.4"W, 1930 m a.s.l.

######## Remarks.

The morphological characters of the Colombian specimens agree with the original description of *E. perarmatus* (Murray, 1907). This species, according to the literature, has a tropical and subtropical distribution; with the type locality, Cape Colony (South Africa), it has also been recorded in Indonesia, Hawaii, United States, and Venezuela (McInnes 1994). The apparent wide distribution we suggest indicates *E. perarmatus* might be a species complex. Therefore further work with original material, or specimens from the type locality, would be required to solve this problem.

This is the first record (*sensu lato*) for Colombia.

###### Genus: *Bryodelphax* Thulin, 1928

####### Bryodelphax kristenseni sp. n.

Taxon classificationAnimalia EchiniscoideaEchiniscidae

http://zoobank.org/87BA6532-6911-4C82-8FA0-689DD1080116

**Figure 1.**
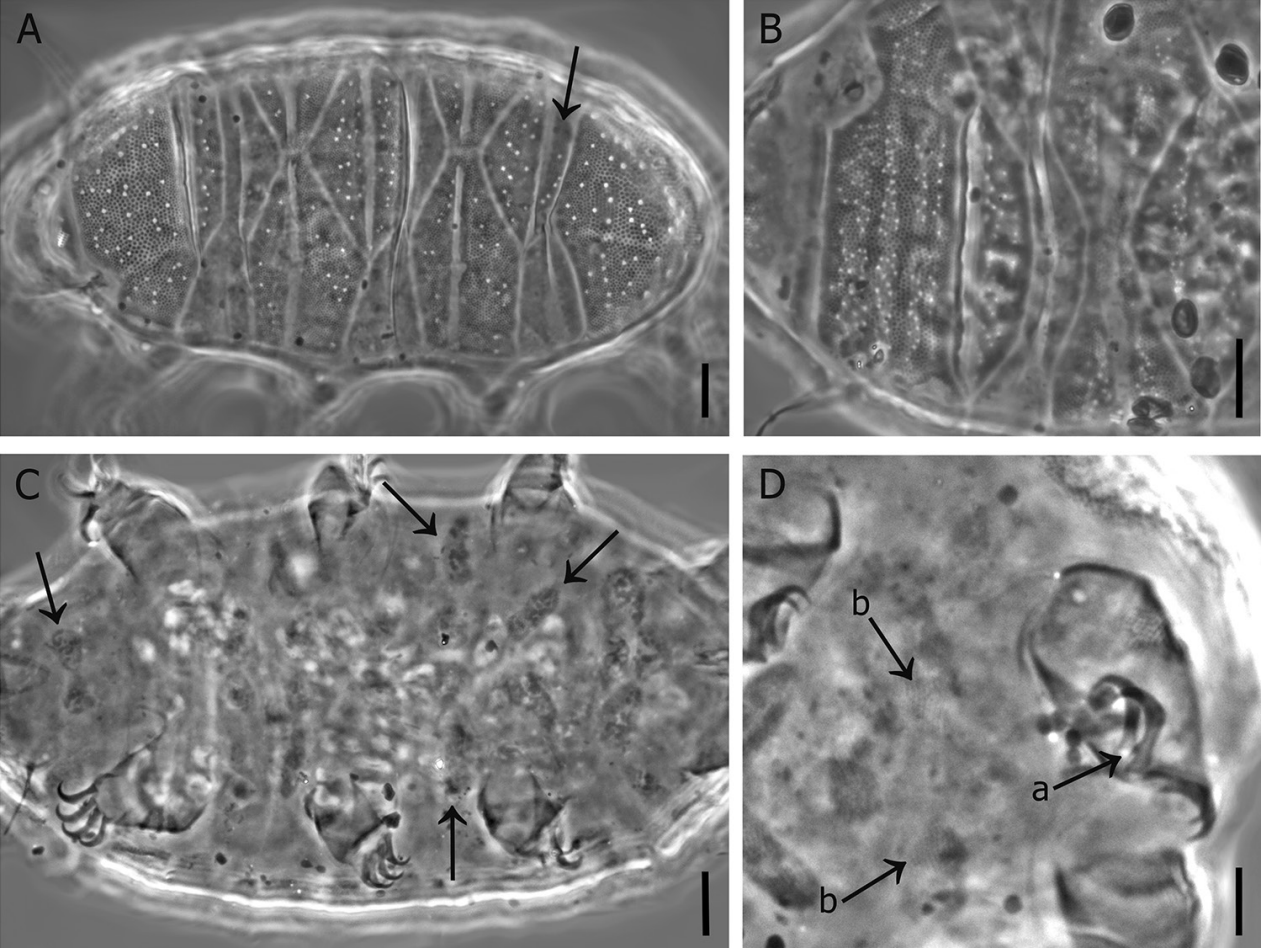
*Bryodelphax kristenseni* sp. n. **A** habitus and dorsal cuticular plates (paratype CBUMAG:TAR: 00137-2; the arrow indicates the caudal portion of the third median plate) **B** scapular plate of the holotype, the ornamentation is visible (the specimen is oriented with the head pointing left) **C** ventral surface of the holotype, with several ventral plates visible (arrows indicate those that are better visible) **D** internal claw spurs (arrow **a**), and granular sculpture of the ventral plates (arrows **b**) (paratype CBUMAG:TAR:00144-7). Scale bars: 10 µm (**A–C**); 5 µm (**D**).

**Figure 2.**
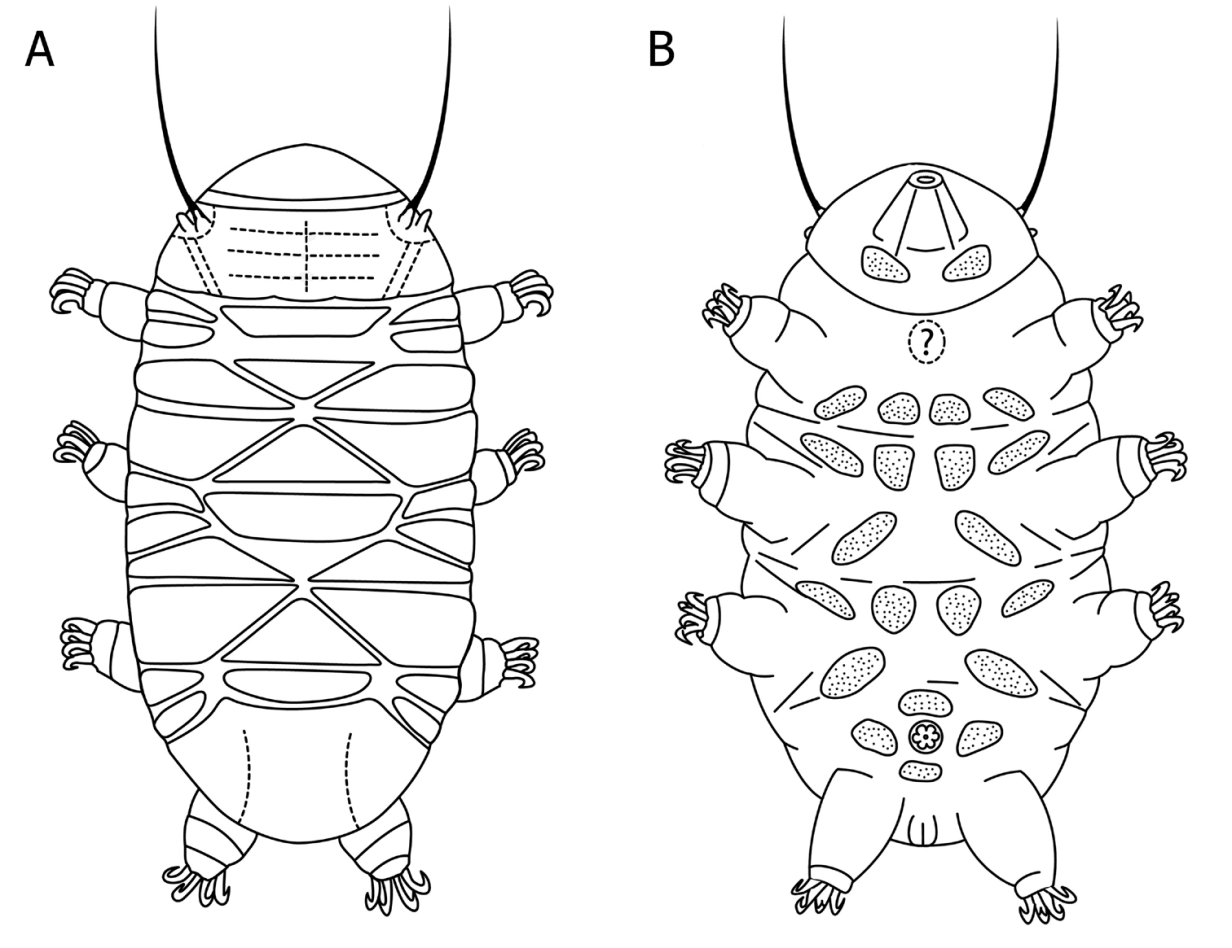
Drawings of the dorsal **A** and ventral **B** plates arrangement of *Bryodelphax kristenseni* sp. n.

**Figure 3.**
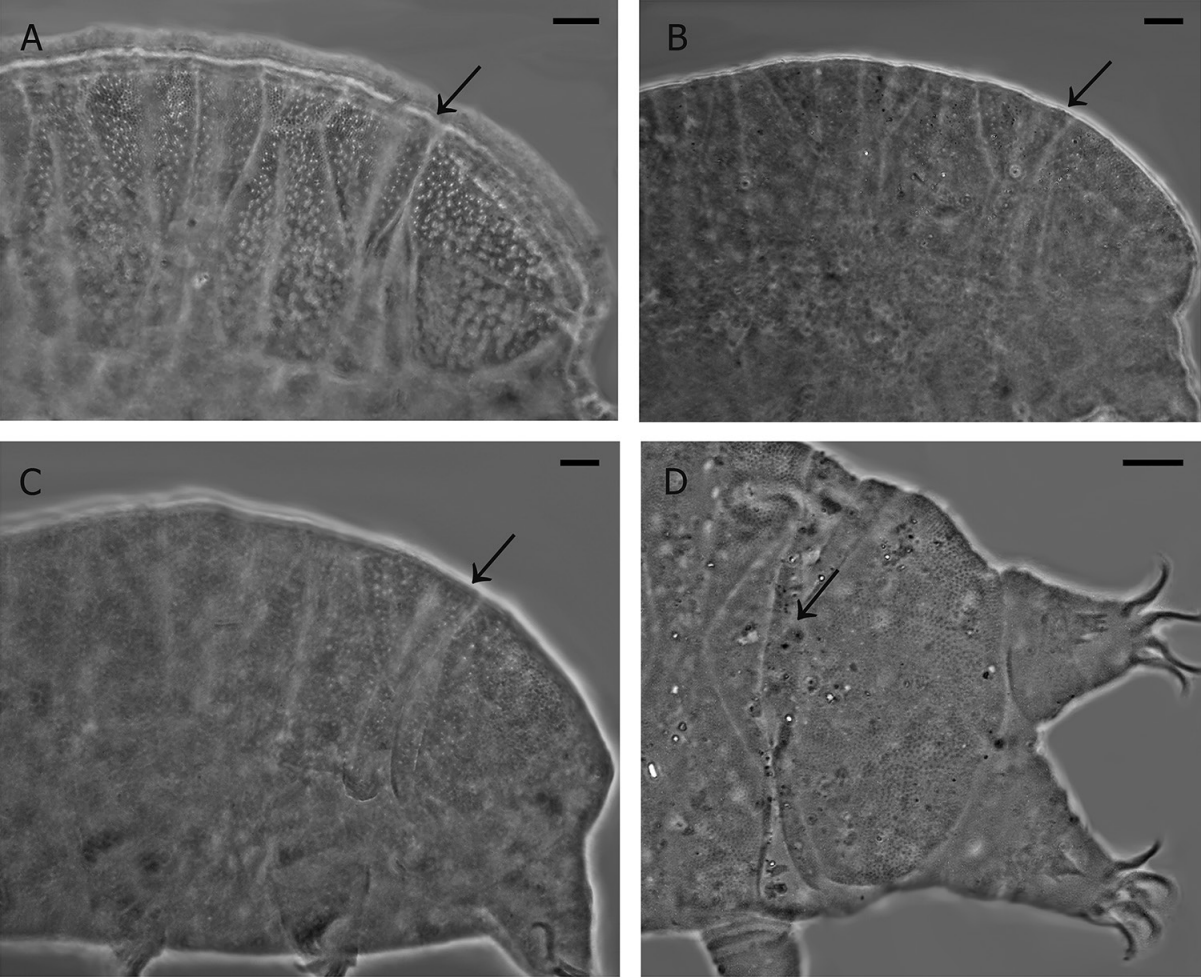
Divided third median plate (arrows indicate its caudal piece) in **A** *Bryodelphax brevidentatus* Kaczmarek, Michalczyk & Degma, 2005 **B** *B. meronensis* Pilato, Lisi & Binda, 2010 **C** *B. parvulus* Thulin, 1928, and **D** *B. weglarskae* (Pilato, 1972). Scale bars 10 µm.

Figs 1 , 2 , 3 , Table 1

######## Material examined.

Holotype and 10 paratypes extracted from a sample composed of lichen (*Parmotrema*), liverworts (*Frullania*, *Plagiochila*), and mosses (Calymperaceae, Amblystegiaceae). The sample was collected in 2011 at the medium basin of Garupal River, Sierra Nevada de Santa Marta, 10°13'48.4"N, 073°48'01.5"W, 543 m a.s.l.

Four additional specimens, CBUMAG:TAR:00198 (1 specimen), 00100 (3 specimens), collected in 2012 from Bella Vista, Sierra Nevada de Santa Marta, 11°05'47.8"N, 74°05'04.4"W, 1,930 m a.s.l. The microhabitat was a lichen from the genus *Usnea*.

*Type repository*: The holotype and paratypes are deposited in the Centro de Colecciones Biológicas de la Universidad del Magdalena (CBUMAG), Santa Marta, Colombia. Slide numbers: Holotype (mature female) CBUMAG:TAR:00143-8; Paratypes: CBUMAG:TAR:00137 (3 specimens: 1 juvenile and 2 mature females), 00138 (5 specimens: 1 larva and 4 mature females), 00143 (1 specimen: mature female), 00144 (1 specimen: juvenile).

######## Diagnosis.

Small *Bryodelphax* with ten paired plus two or three unpaired ventral plates (IX/X:2-(1)-4-4-2-4-2-1-2-1 according to Kaczmarek et al. 2012), often poorly visible, with fine granular sculpture; dorsal plate ornamentation with superficial irregular pores, and deeper dark “dots” (i.e. cuticular pillars); 6 pairs of faint supplementary plates present laterally between paired plates, ventral cuticle between ventral plates smooth; spine on anterior legs and papilla on hind legs absent or not visible in optical microscopy, dentate collar absent.

######## Description of the holotype.

Body colourless, eyespots absent or not visible after mounting. Total body length, 126.5 µm. Scapular and terminal plate not distinctly divided but unsculptured folds indicate the different portions of the plates (Fig. 1B, 2A). In particular on the scapular plate, a pair of lateral longitudinal grooves differentiates clearly the small lateral portions from the main median, which shows a median longitudinal fold crossed by three less defined transversal bends (Fig. 2A). A median longitudinal fold, not always obvious, is also present in the unpaired plates. The terminal plate has a pair of longitudinal folds (Fig. 2A), which separate the plate into a median and two lateral portions; the former appearing crossed by irregular transversal folds that are not always clearly visible (Fig. 1A, 2A). All three median plates divided, but the posterior portion of the third plate is narrow and rectangular. The first median plate transversally subdivided in two parts by a suture devoid of sculpture, a trapezoid anterior portion, and a triangular posterior section with a rounded caudal edge. The anterior portion of the second median plate is triangular and the posterior section trapezoid; in the posterior area an unsculptured triangular region is visible but, due to its appearance, was not interpreted as a plate. The third median plate is divided, the main anterior plate triangular, with an anterior rounded edge, and a posterior sculptured portion, but sometimes hidden by the terminal plate in contracted specimens. Paired plates also divided into an anterior moderately narrower portion, and a posterior wider portion (Figs 1A, 2A). The shape and arrangement of all plates and their sub-portions is outlined in Fig. 2A.

All plates show apparent double sculpture: big pores more or less irregular in size and distribution, often fused to one another (Fig. 1A–B) in some cases almost forming patterns, and a lower level of regular “granules” (i.e. cuticular pillars). The pores tend to form three transversal bands on the scapular plate (Fig. 1B), while on the terminal plate they tend to be grouped in areas that are separated by stripes without pores, thus almost outlining “facets”. On the remaining plates, the pores tend to be arranged on each sub-portion of the plate in a more or less defined transversal band (or a single line on the narrowest sub-plates) (Fig. 1), i.e. a band on the anterior and a band on the posterior portions of the paired plates, a band on the anterior and a single line on the narrow posterior portions of the unpaired plates.

The cuticular pillars (Fig. 1A, B) appear regularly distributed, and vary in size between plates and the different part of each plate/sub-plate. The largest (about 1.2 µm) are on the scapular and terminal plates, and the proportion among the granules of the different plates is as follows: scapular = terminal > posterior portions of the paired plates > anterior portions of the paired plates > unpaired plates. In the latter, the “granules” appear not only smaller but also fainter. On each plate or sub-plate, the terminal plate excluded, the “granules” are larger on the median transverse band, and gradually decrease in the more cephalic and caudal portions as well as the very lateral portions, almost at the borders. On the terminal plate the largest granules lie on a band at about ¾ of the length of the plate, gradually decreasing, going to the more cephalic portion and at the very lateral and caudal portions, almost at the borders. Six pairs of lateral supplementary platelets, difficult to see, are present between the paired plates (Fig. 2A).

Ventral plates present, but faint and difficult to observe, consist of ten paired plus two unpaired (IX/X:2-(1)-4-4-2-4-2-1-2-1 according to Kaczmarek et al. 2012), as depicted in Fig. 2B; the brackets in the formula and the question mark in Fig. 2B indicate the difficulty in ascertaining the presence of an unpaired ventral plate at the level of legs I: if that plate is present, then the unpaired plates are three and not only two, and the plate rows are ten instead of nine. All ventral plates show a faint granulation under PCM (Fig. 1C, arrows). The rest of the ventral cuticle is smooth. All legs with a band of small dots.

Apart from the cephalic cirri, only the lateral filament *A* is present (26.5 µm long = 21.0% of body length and 150.7% of the scapular plate length). Internal cephalic cirrus 8.0 µm long; external cephalic cirrus 11.5 µm long; cephalic papilla 4.0 µm long; clava *c.* 4.7 µm long (Table 1).

Spines on the first pair of legs absent or not visible under PCM. The dentate collar on the fourth pair of legs absent (but the sculptured platelet of the dentate collar present). No papilla visible on the hind legs under PCM. External claws smooth. Internal claws with spur oriented towards the base (Fig. 1D, arrow *a* indicating a spur). Measurements of some structures of the holotype and ranges among the paratypes (larva excluded) are given in Table 1 (Supplementary Data provides all measurements for these specimens). No eggs were found.

**Table 1.** Measurements (µm) and %bo %sc values of selected morphological structures of the hotolotype and paratypes of *Bryodelphax kristenseni* sp. n.

CHARACTER	N	MIN – MAX									MEAN			SD			HOLOTYPE		
		µm			%bo			%sc			µm	%bo	%sc	µm	%bo	%sc	µm	%bo	%sc
Body length	10	106	–	131							121.1			9.1			126.5		
Scapular plate length	10	16.2	–	21.7	13.9	–	19.4				20.4	16.4		1.9	1.5		17.6	13.9	
Lateral appendage A	8	24.8	–	27.2	18.8	–	24.0	115.9	–	163.5	26.5	21.4	129.0	0.8	1.8	15.9	26.5	21.0	150.7
Clava	5	3.8	–	4.8	3.4	–	4.3	15.7	–	26.3	4.7	3.7	19.0	0.4	0.3	4.0	4.7	3.7	21.0
Int. cephalic cirrus	9	6.6	–	8.3	5.2	–	7.0	31.9	–	45.5	8.0	6.3	39.0	0.7	0.5	4.0	8.0	6.3	45.5
Ext. cephalic cirrus	10	9.5	–	13.1	8.5	–	10.6	51.7	–	65.4	11.4	9.2	56.3	1.0	0.7	4.7	11.5	9.1	65.4
Cephalic papilla	7	3.5	–	4.5	3.1	–	3.8	19.3	–	25.6	4.1	3.4	21.2	0.4	0.2	2.2	4.0	3.2	22.8
Ext. claw I	9	5.3	–	6.4	4.7	–	5.6	28.6	–	34.2	6.2	4.9	29.9	0.4	0.3	2.1	6.0	4.7	33.8
Int. claw I	8	5.6	–	7.0	5.0	–	5.9	30.4	–	36.2	6.6	5.3	32.2	0.5	0.3	2.2	6.4	5.0	36.2
Spur	8	1.0	–	1.3							1.2			0.1			1.1		
Spur/Claw		0.2	–	0.2							0.2			0.0			0.2		
Ext. claw II	7	5.1	–	6.3	4.5	–	5.6	27.3	–	32.9	5.8	4.7	28.7	0.4	0.4	1.9	5.8	4.6	32.9
Int. claw II	6	5.5	–	6.6	4.7	–	5.1	23.7	–	31.7	6.0	5.0	26.2	0.5	0.1	3.0	6.0	4.7	26.9
Spur	7	0.9	–	1.1							1.1			0.1			1.0		
Spur/Claw		0.1	–	0.2							0.2			0.0			0.2		
Ext. claw III	7	4.4	–	6.3	4.2	–	5.5	25.0	–	31.7	6.0	4.7	28.5	0.7	0.4	2.2	5.6	4.4	31.7
Int. claw III	4	5.2	–	6.4	4.7	–	5.7	29.1	–	33.6	6.1	4.8	29.7	0.6	0.5	2.1	5.9	4.7	33.6
Spur	4	1.0	–	1.2							1.1			0.1			1.1		
Spur/Claw		0.2	–	0.2							0.2			0.0			0.2		
Ext. claw IV	6	5.2	–	7.2	4.9	–	6.0	29.1	–	35.2	6.5	5.3	31.5	0.7	0.4	2.1			
Int. claw IV	3	6.0	–	6.5	5.0	–	5.5	30.6	–	37.3	6.3	5.3	32.3	0.3	0.3	3.5			
Spur	2	1.0	–	1.3							1.2			0.2					
Spur/Claw		0.2	–	0.2							0.2								

######## Remarks.

The paratypes exhibit the same morphology, but with a certain degree of variation with regard to appearance of the bands of the scapular and terminal plates, more visible in less relaxed specimens. The narrow posterior portions of the median plates (especially with regard to the third), are more visible in well-relaxed specimens but can be totally hidden in contracted specimens. In such specimens, the posterior elements of each couple of supplementary platelets, especially the third, could also be hidden. In addition, the orientation of the specimen on the slide meant the supplementary lateral platelets were not always clearly visible. With regard to the ventral plates, these were evident in some specimens, e.g. that chosen as holotype, but were not always easy to see. In general, the ventral plates varied from faint to almost invisible (without clear indication of a link with life stage); it took a very accurate, long observation under both PCM and DIC to identify all the plates and to be certain of the number and arrangement. Such plates show in PCM a faint granulation, which is actually what, in some cases, made them visible; their borders often being unclear. In some of the specimens not even one ventral plate was apparent at first sight, requiring very careful observation to detect at least some of them; thus this character can pass unnoticed. We therefore recommend great care in observing *Bryodelphax* before considering whether a specimen is without ventral plates, and also without supplementary lateral platelets.

Another character, for which considerable individual variability is noted, is the distribution of the cuticular pores, which may be arranged from a relatively regular distribution, as described in the holotype, to a quite random distribution. Additionally, the transversal bands of pores of the sub-portions of the paired and unpaired plates can be reduced to a single, more or less regular row.

Finally, the papilla of the hind legs in most specimens was not visible, but in a couple of individuals, there appeared to be an extremely small, faint papilla. However, the presence of particles in the slides preparation prevented us from being sure that what we observed was a papilla and not some out of focus particle. This character, therefore, needs to be confirmed.

######## Etymology.

The species is named in honour of Professor Reinhardt Møbjerg Kristensen, in particular for his valuable contribution to the taxonomy of Echiniscidae (Kristensen 1987).

######## Differential diagnosis.

According to Kristensen et al. (2010), the new species falls into the *weglarskae* group due to the presence of ventral plates. Within this group the dentate collar is absent from only two species: *Bryodelphax sinensis* (Pilato, 1974) and *B. aaseae*. However, due to the difficulty in observing the ventral plates in some specimens of our new species, we also compare species descriptions where ventral plates were not reported (stressing the fact that especially in early publications the ventral plates may have passed unnoticed or not considered a valuable character). Species with the same type of cuticular ornamentation, and without the dentate collar include: *B. parvulus*, *B. asiaticus* Kaczmarek & Michalczyk, 2004 and *B. ortholineatus* (Bartoš, 1963).

*Bryodelphax aaseae* (Kristensen, Michalczyk & Kaczmarek, 2010) is the most similar species, sharing with the new species the same ventral plate configuration (if the median unpaired plate at the level of legs I is also present in the new species). These plates were described as smooth by the authors, but in Kristensen et al. (2010 – figs 9–11 and 19), there is the appearance under PCM of granulation, and this might be similar to the new species. Another character we noted was the apparent presence of lateral supplementary platelets between the paired plates in *B. aaseae*, (see: Kristensen et al. 2010 – fig. 7), which was not mentioned by the authors. Despite the similarities, *B. kristenseni* sp. n. differs from *B. aaseae* by: less evident ventral plates; the unpaired ventral plate at the level of the pharyngeal bulb appears absent in *B. kristenseni* sp. n. (present in *B. aaseae*); shorter cirrus A (18.8–24.0% of the body length *vs.* about 24–34% µm in *B. aaseae*); longer clava (3.4–4.3% of the body length *vs.* less than 3% µm in *B. aaseae*); shorter claws (4.2–6.0% of the body length *vs.* about 7.3–7.7% in *B. aaseae*).

*Bryodelphax kristenseni* sp. n. differs from *B. sinensis* by having supplementary platelets, more numerous ventral plates (IX/X:2-(1)-4-4-2-4-2-1-2-1 *vs* VII:2-2-2-2-2-2-1 in *B. sinensis*), ventral cuticle smooth (dotted in *B. sinensis*), longer clava (3.4–4.3% of the body length *vs.* about 2.5% in *B. sinensis*).

The diagnosis of *B. parvulus* was revised by Pilato et al. (2010), and this species should lack ventral plates. Moreover, another difference with the new species is the length of the clava: 3.4–4.3% of the body *vs.* about 2.5% of the body in *B. parvulus* (see Pilato et al. 2010, in which a specimen from Poland attributed to *B. parvulus* by Węglarska 1959, and confirmed by Pilato, was measured, slide No. 1476 of Pilato and Binda collection).

*Bryodelphax kristenseni* sp. n. differs from *B. asiaticus*, which lacks the ventral plates, in having non-granulated ventral cuticle, anterior portions of median plates 1 and 2 markedly larger than the posterior portions (which are almost a stripe), while in *B. asiaticus* the posterior portions of those plates are only slightly smaller.

The new species differs from *B. ortholineatus* in having spurs on internal claws (absent in *B. ortholineatus*), in having supplementary platelets (not mentioned in the original description (Bartoš, 1963) and reported as absent by Fontoura et al. 2008), and the shape of the median plates looks different in Bartoš’ original drawing, but it must be stressed that the drawing was very stylised.

## Discussion

As mentioned above, in *B. kristenseni* sp. n. the third median plate is divided into an anterior and a posterior portion; this character, until now, has been considered typical of the genus *Bryochoerus*, while in *Bryodelphax* the third median plate has been considered undivided.

At first, there were doubts on the identification due to the division of the third median plate, although hidden in some specimens, which led us to *Bryochoerus*; on the other hand, there was evident resemblance between these specimens and *Bryodelphax aaseae*, and with other species, e.g., *Bryodelphax weglarskae* (Pilato, 1972). This encouraged careful examination of that species as well as many others congeners, and it was noted that the divided third median plate was also present in *B. aaseae* (figs 7 and 8 in Kristensen et al. 2010), *B. asiaticus* (fig. 6 in Kaczmarek and Michalczyk 2004), *B. parvulus* (Ramazzotti and Maucci 1983, page 221, fig. 70 from Thulin’s 1928 original description), and the following material from the Pilato and Binda collection, which was examined: *B. brevidentatus* (paratype, slide No. 5386, Fig. 3 A), *B. meronensis* (holotype, slide No. 5350, Fig. 3 B), *B. parvulus* (from Israel, slide No. 5348, Fig. 3C; from Northern Italy, slide Nos. 1288, 1290–91; from Morocco, slide No. 1280; from central Sicily, slide No. 1880, and from Ustica Island, about 60 km North from Sicily, slide No. 1296). Furthermore, a specimen of *B. weglarskae* also had that piece of plate partially covered by the terminal plate (Fig. 3 D).

The definition of the genus *Bryodelphax* is therefore suggested as follows:

Small Echiniscidae with non-flexible buccal tube with CaCO_3_ encrusted stylet supports. No lateral or dorsal appendages present except cirrus *A.* Median plates all divided, but the caudal portion of the third median plate may be hidden by the terminal plate. Without pseudosegmental plates. Ventral plates may be present.

With respect to Kristensen’s (1987) definition, we have preferred not to include the presence of “red granulate eyes” as a character of the genus because the presence or absence of eyes is variable in many genera, and can be lost in the slide mounting process (e.g. *B. kristenseni* sp. n.).

## Conclusions

Authors from an earlier period (*ca.* 1900–1950s) traditionally considered many species to be cosmopolitan, which in tardigrade taxonomy created species-groups. These species-groups, along with past errors and misinterpretations, now require careful analysis in order to amend taxa descriptions and differentiate the sibling species. As taxonomic knowledge has progressed, key characters have been added that were not considered essential in older references. We are left with a legacy of early species descriptions that are often impossible to identify without type material, which in many cases is sadly unavailable. Taking into account the absent or poor state of older type material, and the difficulty in resampling a vaguely described *locus typicus*, the possibility of abolishing or classifying a suspect species as “*species dubia*” should be considered. This would help prevent further confusion created by non-taxonomists or beginners using an apparently simple diagnostic key (e.g., Ramazzotti and Maucci (1983) monograph) to record questionable species.

With our present contribution, based on material in a museum collection, three new records enrich the list of Echiniscidae recorded for Colombia. In addition, a species new to science was discovered, which provided the occasion to make evaluations at a higher taxonomic level. The fact that a relatively limited study provides new and interesting results, in spite of the great efforts from past decades, is evidence of how little is known about tardigrade fauna and biogeography for most regions of the world. It further highlights how much our taxonomic knowledge has grown and can still grow.

## Supplementary Material

XML Treatment for Echiniscus dariae

XML Treatment for Echiniscus kofordi

XML Treatment for Echiniscus perarmatus

XML Treatment for Bryodelphax kristenseni
